# Hybrid sorghum breeding in China: A historical review and perspectives

**DOI:** 10.1111/jipb.70047

**Published:** 2025-09-26

**Authors:** Xiangxiang Meng, Lu Li, Qian Qian, Liang Jiang, Zhaosheng Kong

**Affiliations:** ^1^ Shanxi Hou Ji Laboratory, College of Agriculture Shanxi Agricultural University Taiyuan 030031 China; ^2^ Yazhouwan National Laboratory Sanya 572024 China

**Keywords:** agricultural innovation, CMS, dwarf breeding, hybrid sorghum breeding, sorghum breeding history

## Abstract

Sorghum (*Sorghum bicolor* (L.) Moench) is a climate‐resilient C_4_ cereal and a vital pillar of food and feed security in arid and semi‐arid regions worldwide. In China, the development and widespread adoption of hybrid sorghum breeding have revolutionized the crop's productivity, playing a transformative role in enhancing both yield and quality. The success of hybrid sorghum, particularly through the utilization of cytoplasmic male‐sterility (CMS) systems, has marked a milestone in agricultural innovation, enabling the large‐scale production of high‐performing hybrids. The implementation of dwarf breeding and the continuous renewals of sorghum hybrid varieties have been pivotal in driving these improvements. As we commemorate the 60th anniversary of the promotion and application of three‐line hybrid sorghum, we recognize the groundbreaking contributions of Chinese researchers in advancing sorghum breeding science. This review highlights key scientific breakthroughs and systematically summarizes the evolution of sorghum breeding in China. By reflecting on both past achievements and prospective opportunities, we aim to inform strategies that will sustain and enhance sorghum's contribution to China's agricultural resilience and global food security.

## Graphical abstract

To commemorate the 60th anniversary of the promotion and application of three‐line hybrid sorghum in China, this review highlights pivotal scientific breakthroughs, systematically summarizes the progress in hybrid sorghum breeding and dwarf sorghum breeding in the country, and presents an outlook on the future of sorghum breeding.

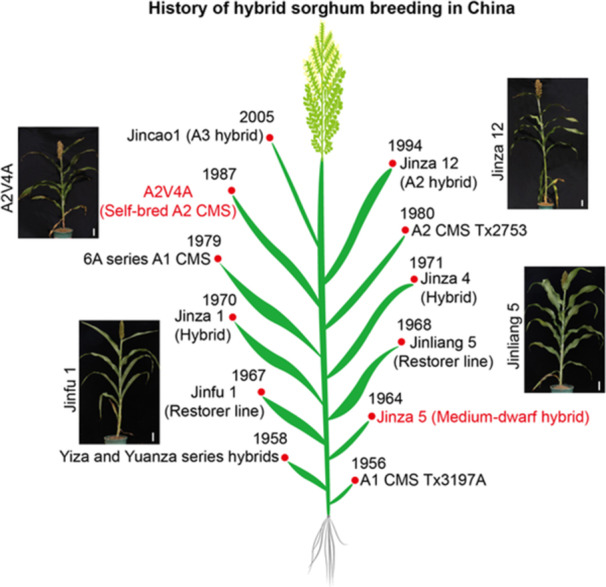

## INTRODUCTION

Sorghum (*Sorghum bicolor* (L.) Moench), recognized as the fifth most significant cereal crop worldwide, serves as an adaptable and stress‐tolerant food source for approximately half a billion people across Africa and Asia. It is hypothesized that sorghum was initially domesticated from its wild progenitor (*Sorghum bicolor* ssp*. verticilliflorum*) in Ethiopia and Sudan over 5,000 years ago (Clark and Stemler, 1975; Wendorf et al., 1992). Characterized by high‐content protein and fiber, sorghum stands out as a multipurpose crop with extensive applications across diverse industries, including food production, animal feed, fermentation, green energy generation, and traditional broom manufacturing. Given its wide‐ranging utility, enhancing both the yield and nutrient quality of sorghum through breeding programs becomes a crucial imperative.

Sorghum has a cultivation history of at least 4,000–5,000 years in China. Since the Ming and Qing dynasties, sorghum has gradually become one of the staple foods in northern China. In 1952, the cultivation area of sorghum in China reached 9.33 million hectares (ha), with an average yield of 1,185 kg/ha. By 2022, due to the replacement by rice and maize, the cultivation area of sorghum had decreased significantly to 674,500 ha. However, its yield achieved a significant increase, reaching 8,900 kg/ha (Wang and Zhao, 2024; Chen et al., 2025). Despite the sharp decline in the sorghum cultivation area over the past seven decades, sorghum played a crucial role in alleviating food shortages in North China before the 1970s. In the mid 1960s, under the collaborative efforts of Professor Tiantang Niu and his colleagues, dwarf hybrid breeding of sorghum was initiated in China, which triggered the large‐scale promotion and planting of hybrid sorghum in the country. The application of heterosis in sorghum and the promotion of hybrid sorghum in China have significantly improved sorghum yields and driven the development of the sorghum industry in China.

## HYBRID SORGHUM BREEDING: AN ENGINE FOR THE BOOST OF SORGHUM PRODUCTION IN CHINA

### Development history of hybrid sorghum breeding in China

**Figure 1:**
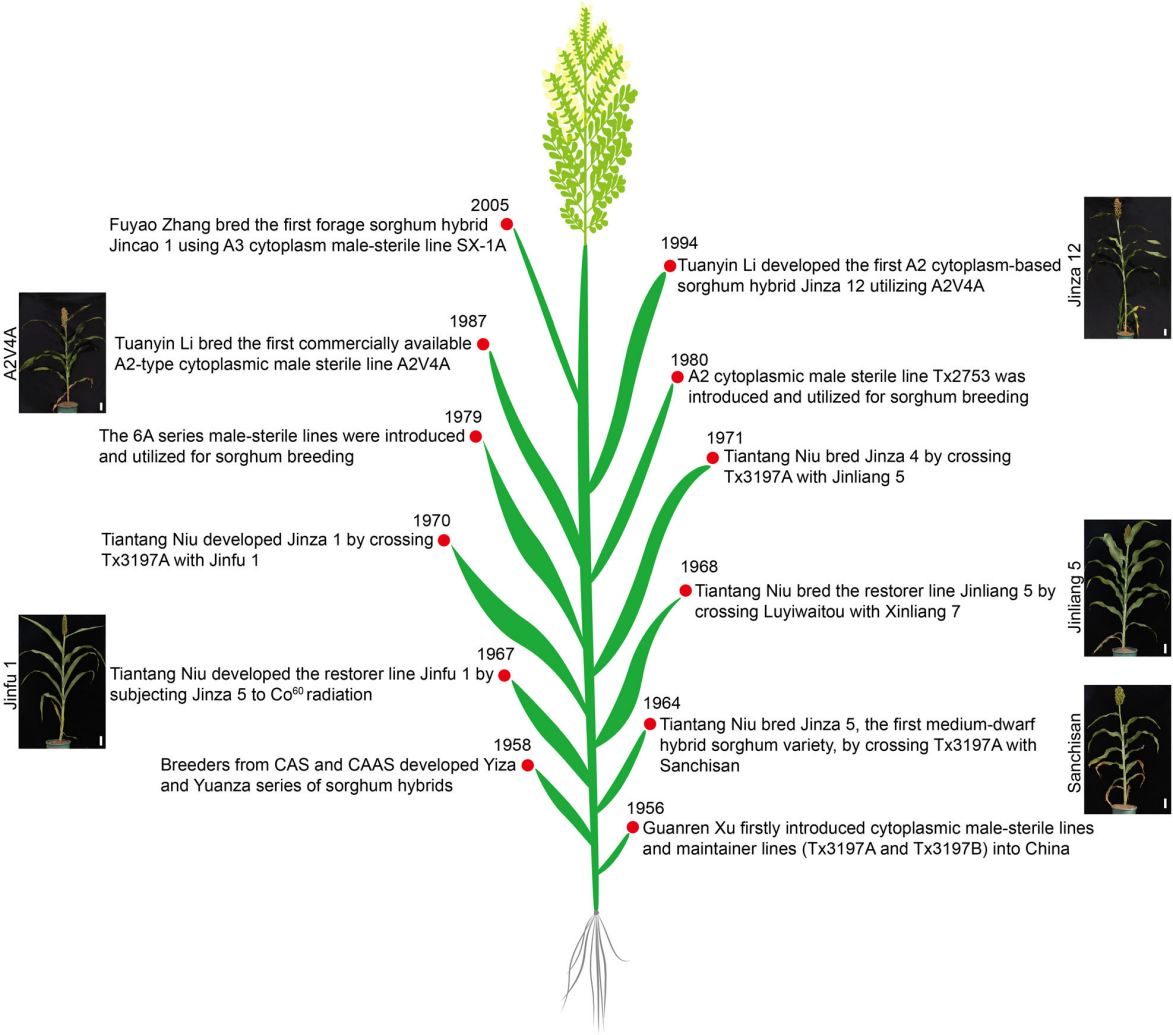
History of three‐line hybrid sorghum breeding and dwarf sorghum breeding in China The available photographs of the representative sorghum varieties have been provided in combination with the historic route. Scale bars represent 10 cm.

Modern sorghum breeding in China was initiated in the 1920s and can be categorized into three distinct periods: (1) collection, evaluation and promotion of local varieties; (2) conventional variety breeding through methods such as selection breeding and cross‐breeding; and (3) utilization of heterosis through the development of single‐cross hybrids from inbred parents (Yang, 1997). Since the 1960s, heterosis breeding has emerged as the primary approach in Chinese sorghum breeding, significantly enhancing yields and improving resistance to both abiotic and biotic stresses. In 1954, Stephens and Holland discovered the “milo” or A1 cytoplasm and harnessed cytoplasmic male sterility (CMS) to produce hybrid sorghum seeds (Stephens and Holland, 1954). This breakthrough paved the way for large‐scale hybrid sorghum production (Figure 1). In 1956, Guanren Xu introduced the male sterile line Tx3197A and the maintainer line Tx3197B from the United States into China (Yan et al., 2024). Building on this genetic material, the breeders from the Chinese Academy of Sciences (CAS) and the Chinese Academy of Agricultural Sciences (CAAS) successfully bred the Yiza and Yuanza series of hybrids in 1958. Although these early hybrids boosted grain yield by 20%–60%, reaching an average of 2,000 kg/ha, they were not widely adopted. Their excessive height and weak stems made them prone to lodging, resulting in subsequent yield losses (Reddy and Reddy, 2019).

In 1964, Tiantang Niu from Shanxi Academy of Agricultural Science (SAAS) developed the first high‐yield medium‐dwarf sorghum hybrid, Jinza 5, by crossing Tx3197A with Sanchisan (Figure 1). The yield of Jinza 5 sorghum was doubled, making a significant contribution to solving the food shortage problem of that era. Utilizing Jinliang 5 and Jinfu 1, Tiantang Niu developed sorghum hybrids Jinza 4 and Jinza 1, respectively, in the early 1970s. Owing to their high yields and lodging tolerance, the cumulative planting area of these three hybrids exceeded 7.06 million ha, spurring the first wave replacement of sorghum hybrids in China. Meanwhile, numerous sorghum hybrid varieties such as Tieza 6, Shenza 3, and Jinza 75 were released by different breeders, contributing to an average increase in sorghum yield to 6,600 kg/ha by the mid 1970s. In 1979, the 6A series male sterile lines such as Tx622A, Tx623A, and Tx624A were introduced from the United States (Gao et al., 2010) (Figure 1). Among them, the hybridization of Tx623A with Jinliang 5, Jinfu 1 and their derivative lines realized the renewal of sorghum hybrids in China. Thereafter, in the 1980s, the representative hybrid sorghum varieties such as Liaoza 1, kangsi, Jinzhong 405, Jinza 11, and Aoza 1 were successionally released with an average yield reaching 7,300 kg/ha. In the 21st century, with the development of hybrid breeding technology, the yield of hybrid sorghum has reached 8,900 kg/ha in China (Chen et al., 2025).

In addition to the A1 CMS, other types have also played an important role in the development process of hybrid sorghum. It has been proposed that, compared with the A1 CMS lines, A2 CMS lines have stronger anti‐abortion and stress‐resistance capabilities (Zhang et al., 2005). The initial A2 CMS line, A2 Tx2753, was developed by Schertz from the United States (Schertz, 1977) and introduced into China in 1980. Subsequently, Chinese sorghum breeders utilized the A2 cytoplasm as the sterile source and successfully developed A2 CMS lines such as A2V4A, SX44A, 7050A and 2055A. During the 1990s, a series of hybrid sorghum varieties derived from A2 cytoplasm emerged, including Jinza 12, Liaoza 10, Jiza 80, Jiza 83, and Siza 25. In this period, a number of self‐bred sterile lines were extensively applied in breeding and production processes. As a result, the era of relying on the import of sorghum sterile lines from the United States was essentially terminated. Notably, in 1987, the breeder Tuanyin Li from SAAS cultivated the first commercially available A2‐type CMS line (A2V4A) in the world by crossing and backcrossing A2 TAM428 with V4 (picklet). And then Tuanyin Li developed Jinza 12 in 1994 utilizing A2V4A, which is the first sorghum hybrid based on A2 cytoplasm in China (Li et al., 1999) (Figure 1). This achievement earned him the third prize of the National Invention Award in 1998. The successive development of A2‐type hybrid sorghum varieties and their promotion nationwide have greatly driven the upgrade of hybrid sorghum in China.

In China, sweet sorghum is mainly grown for silage and thus requires high biomass. Sweet sorghum breeding in China started relatively late, with the introduction of improved sweet sorghum varieties such as Rio, Roma and Wray from abroad beginning in the 1970s to 1980s. Liaoning Academy of Agricultural Science (LAAS) bred the hybrid sweet sorghum varieties Liaosiza 1 and Liaosiza 2 in 1989 and 1995, respectively, using CMS (Reddy and Reddy, 2019). Subsequently, Chinese research institutions bred a number of excellent and high‐yielding sweet sorghum varieties. For example, the Institute of Genetics and Developmental Biology (IGDB), and the Institute of Botany (IB) in CAS developed the “Zhongketian” series and “Ketian” series of sweet sorghum varieties, respectively (Li et al., 2015; Jing et al., 2018). Meanwhile, the hybrid sweet sorghum varieties such as Liaotian 1, Liaotian 2 and Nengsi 2 have also been released (Hou et al., 2012; Reddy and Reddy, 2019). A3 cytoplasm, which demonstrates strong anti‐abortion capability, has been challenging to restore its fertility in breeding (Zhang et al., 2005). This limitation has hindered its utilization in grain sorghum breeding. However, it can be well applied in the breeding of forage sweet sorghum, as forage sweet sorghum is typically harvested before flowering. The forage cultivar Jincao 1, the first sorghum hybrid in the world based on A3 cytoplasm, was bred by Fuyao Zhang from SAAS by crossing A3 SX‐1A with sudangrass. After two harvests, the fresh grass yield of Jincao 1 reached 150,792 kg/ha (Zhang et al., 2005). To date, Fuyao Zhang and Junai Ping have developed 13 forage sweet cultivars using A3 CMS lines. These include Jincao 1–Jincao 9 and Jinmu 1–Jinmu 4. Furthermore, breeders from LAAS have also bred hybrid sweet sorghum varieties such as Liaotian 10 and Liaotian 13 using A3 cytoplasm (Wang et al., 2014a, 2014b). Due to insufficient investment and attention, the number of sweet sorghum varieties bred in China remains small, and the development of sweet sorghum hybrids requires more input from the government and breeders.

### Mechanisms of three‐line hybrid in sorghum

**Figure 2:**
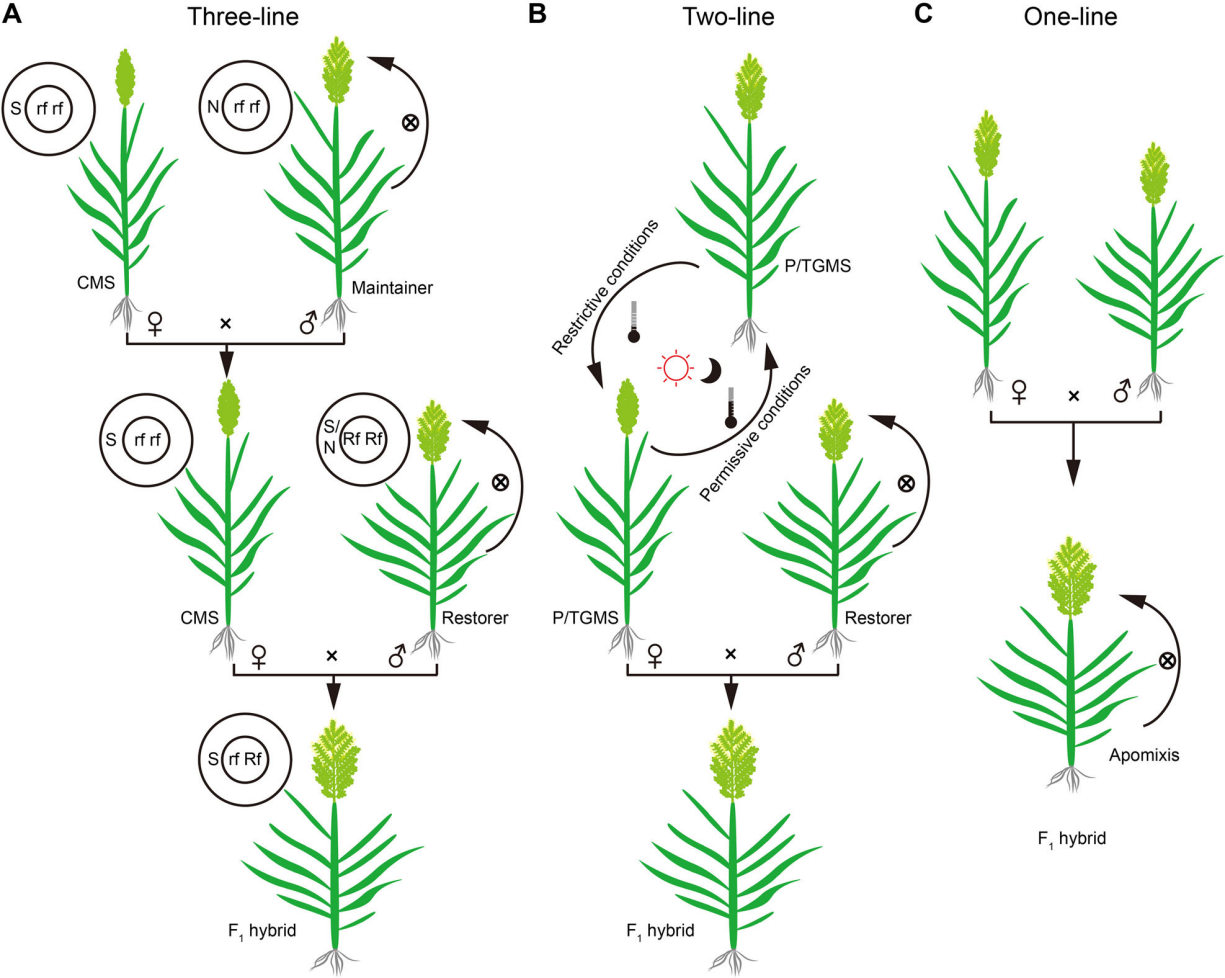
Hybrid sorghum breeding using the three‐, two‐, and one‐line methods **(A)** Three‐line method. **(B)** Two‐line method. **(C)** One‐line method. CMS, cytoplasmic male‐sterility; N, normal cytoplasm; P/TGMS, photoperiod‐/thermo‐sensitive genic male sterility; Rf, restorer‐of‐fertility; S, abortive cytoplasm; × represents hybridization; ⊗ represents self‐pollination; ♀ represents female parents; ♂ represents male parents.

The three‐line breeding system employs CMS lines for hybrid development, where the CMS female parent (A‐line) is pollinated by a maintainer (B‐line) to perpetuate male sterility, while a fertility restorer line (R‐line) is required to cross with the A‐line to generate fertile hybrids (Rooney, 2004) (Figure 2A). Following the establishment of the A1 (milo) CMS system in 1954 (Stephens and Holland, 1954), subsequent studies identified several non‐milo CMS systems in sorghum, designated as A2, A3, A4, A5, A6, and 9E (Webster and Singh, 1964; Ross and Hackerott, 1972; Schertz and Ritchey, 1978; Rao et al., 1984; Worstell et al., 1984). Despite these advancements, commercial sorghum hybrids globally predominantly utilize A1 cytoplasm, with A2 cytoplasm being the secondary choice (Schertz and Ritchey, 1978; Liu et al., 2000; Reddy et al., 2007). The limited application of non‐milo CMS systems is attributed to challenges such as inconsistent male‐sterility stability, scarcity of elite restorer lines, cytoplasmic influences on agronomic traits, and insufficient heterosis for commercial viability (Moran and Rooney, 2003; Reddy et al., 2005).

**Figure 3:**
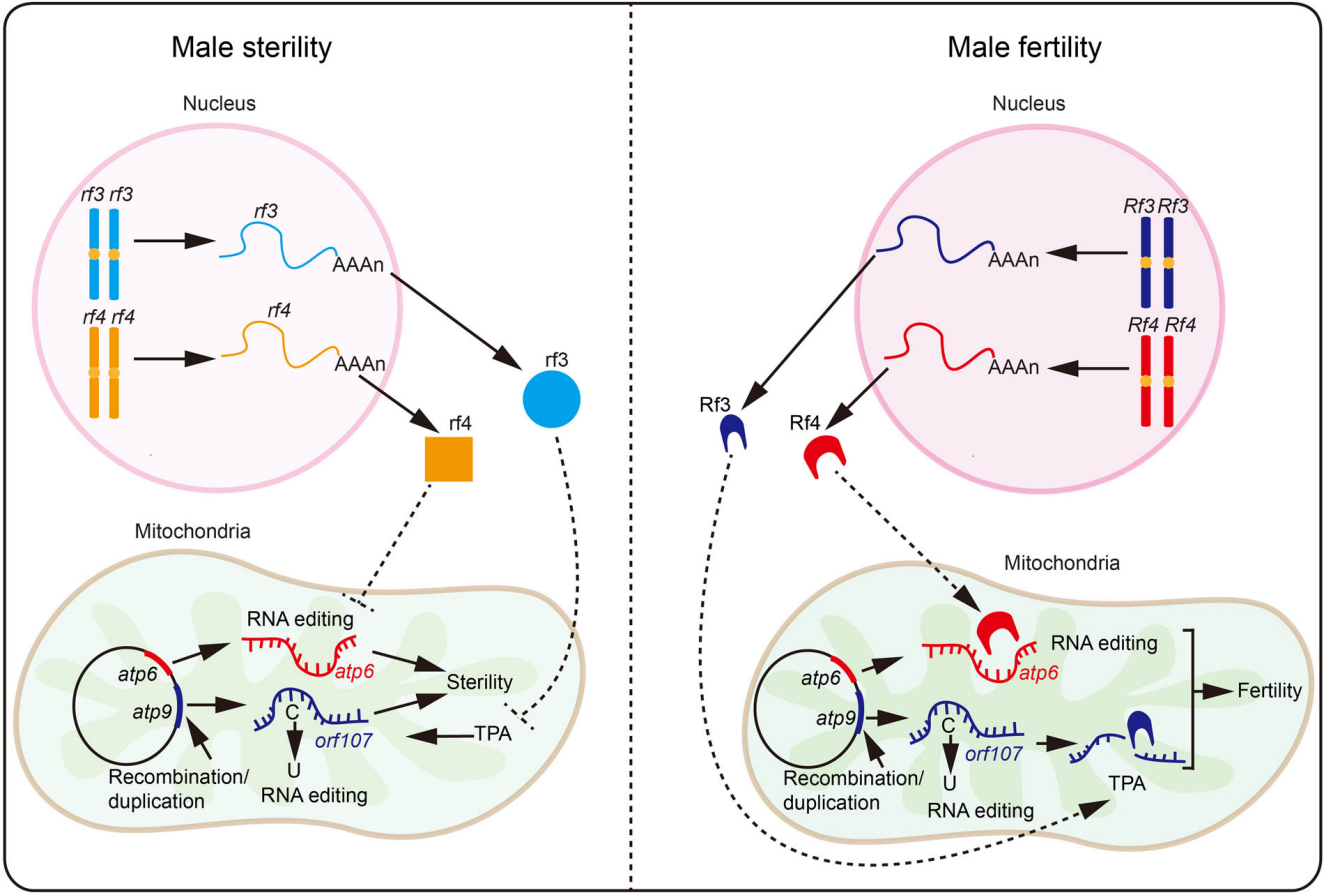
Mechanisms of A3 cytoplasmic male sterility (CMS) and fertility restoration in sorghum The *rf3/rf3* genotype is associated with defective transcript processing activity (TPA) on *orf107*, a gene that results from recombination/duplication with *atp9*, causing male sterility. Alternatively, defective RNA editing of the *atp6* transcript can lead to CMS in the A3 CMS line when the *rf4/rf4* genotype is present. In contrast, the cooperative action of both Rf3 and Rf4, which promotes *orf107* transcript processing and *atp6* transcript editing, respectively, is required to achieve complete fertility restoration of the A3 CMS line. *Rf*, restorer‐of‐fertility; *orf*, open reading frame; TPA, transcript processing activity.

Cytoplasmic male sterility is characterized by the lack of functional pollen production, and it is commonly associated with chimeric mitochondrial open reading frames (ORFs). CMS can be restored by restorer‐of‐fertility (*Rf*) genes encoded in the nucleus. Thus, CMS/Rf systems seem to arise from specific nuclear–mitochondrial interactions (Hanson and Bentolila, 2004; Zhang et al., 2020). In the mitochondrial DNA (mtDNA) of the A3 cytoplasm derived from IS1112C, an *orf* associated with male sterility has been identified and named *orf107*, which results from recombination/duplication with *atp9*. *Orf107* encodes a predicted 11.85 kDa polypeptide and its C‐terminal residues exhibit significant similarity to those of ORF79, a protein linked to CMS in rice. The *orf107* transcript undergoes RNA editing, with four C‐to‐U conversions detected at specific sites (Tang et al., 1996) (Figure 3). Restorer lines capable of restoring fertility to A3 CMS possess transcript processing activity (TPA), which cleaves approximately 75% of full‐length *orf107* transcripts into smaller 380 bp fragments, leading to a substantial reduction of intact *orf107* transcripts (Figure 3). In contrast, CMS lines exhibit negligible TPA (Pring et al., 1998). Genetic studies have demonstrated that TPA is governed by the nuclear restorer gene *Rf3* (Tang et al., 1996; Tang et al., 1998) (Figure 3). Introduction of *Rf3* via hybridization modulates *orf107* transcript processing; however, *Rf3* alone is insufficient to fully restore fertility. Fertility restoration in IS1112C (A3) CMS follows a gametophytic model controlled by two complementary nuclear genes. While Rf3 regulates *orf107* transcript processing, a second gene, *Rf4*, is hypothesized to influence the editing of the mitochondrial *atp6* gene. Defective RNA editing of the *atp6* transcript can lead to CMS (Howad and Kempken, 1997; Howad et al., 1999) (Figure 3). Cooperative action of both *Rf3* and *Rf4* is required to achieve complete fertility restoration in A3 CMS lines (Figure 3). This two‐gene interaction highlights the complex genetic mechanisms underlying mitochondrial‐nuclear crosstalk in plant reproductive biology (Pring et al., 1999). Overall, the mechanism of CMS in sorghum remains largely unexplored.

Nuclear‐encoded *Rf* genes typically counteract CMS by encoding pentatricopeptide repeat (PPR) proteins, which function as sequence‐specific RNA‐binding regulators of mitochondrial transcripts (Dahan and Mireau, 2013). To date, six major *Rf* genes (*Rf1*–*Rf6*) have been characterized in sorghum. *Rf1* (Sobic.008G147400), responsible for restoring fertility in A1 cytoplasm, encodes the PPR13 protein. Comparative analysis revealed a nonsynonymous substitution (S27 to L26) and an arginine insertion (R26) in sterile plants, accompanied by single‐nucleotide polymorphisms (SNPs) and a small insertion and deletion (InDel) in the 5′ region of the PPR13 transcript (Klein et al., 2005). The *Rf2* locus, another major restorer for A1 cytoplasm, was fine mapped to a 10.32‐kb region on chromosome 2 harboring the *PPR* gene Sobic.002G057050. This gene exhibits elevated expression in restorer lines and contains a missense mutation in its first exon (Jordan et al., 2010; Kante et al., 2018; Praveen et al., 2018). Furthermore, *Rf3* and *Rf4*, mapped on chromosome 7, restore fertility in A3 cytoplasm (Tang et al., 1998; Tang and Pring, 2003). *The Rf5 locus*, which restores both A1 and A2 CMS, was initially localized to an ~584‐kb region on chromosome 7, containing seven P‐subfamily *PPR* genes (Jordan et al., 2011). Subsequent fine mapping narrowed this region to 140 kb, with *PPR.4* emerging as the strongest candidate due to sequence homology with the *Rf1* in rice (Kiyosawa et al., 2022). Last, *Rf6* was mapped to a 43‐kb region on chromosome 4, where the *PPR* gene Sobic.004G004100 was identified as the most probable restorer. A frameshift mutation caused by an 11‐bp insertion introduces a premature stop codon (TGA) in CMS lines, disrupting its function (Praveen et al., 2015).

## DWARF GRAIN SORGHUM BREEDING: A CORNERSTONE OF HYBRID SORGHUM SUCCESS IN CHINA

### Development history of dwarf grain sorghum breeding in China

Hybrid sorghum breeding has significantly increased sorghum yield by utilizing heterosis. However, dwarf sorghum breeding has further increased yield by enhancing lodging resistance and planting density on the basis of utilizing heterosis, thus promoting the large‐scale popularization of sorghum in China (Gao et al., 2006). In the 1950s, although early hybrid sorghum significantly increased yields, their excessive plant height (2.5–3.0 m) led to lodging, which caused yield losses and restricted the large‐scale promotion of sorghum (Reddy and Reddy, 2019). Tiantang Niu observed that Sanchisan, a dwarf landrace (~1.2 m) from Shanxi Province, exhibited lodging tolerance after a storm. Leveraging this important finding, in 1964, he developed the first high‐yield medium‐dwarf sorghum hybrid (1.8–2.0 m), Jinza 5, by crossing dwarf female parent Tx3197A with Sanchisan as the restorer line (Figure 1). Jinza 5 combined reduced plant height with enhanced stem strength, facilitating widespread adoption and effectively ushering in large‐scale promotion and planting of hybrid sorghum in China. Meanwhile, Tiantang Niu proposed the “II‐dwarf genotype” breeding strategy, which refers to breeding with sorghum restorer lines that have two homozygous recessive dwarf genes. In 1967, Tiantang Niu bred a restorer line, Jinfu 1 (~1.5 m), by subjecting Jinza 5 to Co^60^ radiation. In 1968, he developed the restorer line Jinliang 5 (~1.7 m) through a cross between Luyiwaitou and Xinliang 7. Utilizing “II‐dwarf genotype” Jinlaing 5 and Jinfu 1, Tiantang Niu developed medium‐dwarf sorghum hybrids Jinza 4 and Jinza 1 (~1.8 m) in early 1970s, respectively. The reduced plant height of the Jinza 5/4/1 has improved the lodging resistance and yield of sorghum significantly, which has greatly promoted the large‐scale popularization of hybrid sorghum. As a result, Tiantang Niu was awarded the National Science Congress Award in 1978. Due to the outstanding agronomic traits, Jinliang 5 and Jinfu 1 emerged as the two flagship restorer lines in Chinese sorghum breeding. These two lines and their derivatives accounted for 45% of the sorghum restorer lines in China (Hou et al., 2000). Given the immense contributions of two outstanding restorer lines to sorghum breeding, Tiantang Niu's team won the third prize of the National Invention Award in 1991. In the 1970s, dwarf breeding and hybrid varieties were widely utilized in sorghum breeding in China (Yan et al., 2024). Subsequently, Chinese breeders independently developed dwarf sorghum male sterile lines such as SX44A (~1.2 m), 7050A (~1.3 m), and 2055A (~0.8 m), breeding a series of excellent dwarf sorghum hybrids (Zou et al., 2010; Li et al., 2011). In the 21st century, the primary breeding goal for sorghum hybrids developed by research institutions across the country has been to create medium‐dwarf varieties typically with a plant height of 1.65–1.8 m such as Jinza 22 (~1.67 m) and Jiza 101 (~1.80 m). After 2008, the breeding of sorghum varieties suitable for mechanized production was the main objective of research institutions. The plant height of these varieties has reduced to 1.0−1.6 m such as Jinza 33 (~1.57 m), Jinza 34 (~1.35 m), and Jinliang 211 (~1.1 m) (Chen et al., 2025).

### Molecular genetic basis of dwarf grain sorghum breeding

Dwarfism serves as a cornerstone trait in modern agriculture, conferring lodging resistance and enabling mechanized harvesting. Current molecular evidence identifies four major loci (*Dw1*–*Dw4*) controlling sorghum plant height through independent genetic mechanisms (Quinby and Karper, 1954). Recessive mutations of each locus lead to decreases in plant height. It has been revealed that the key male sterile lines for sorghum primarily belong to the “III‐dwarf genotype” varieties from the Kafir subspecies and its improved derivatives, such as Tx3197A, which harbors three recessive homozygous dwarf alleles (genotype *dw1Dw2dw3dw4*). The restorer line Sanchisan carries solely the *dw4* dwarf gene (*Dw1Dw2Dw3dw4*) (Wang et al., 2024b), with homozygosity at this locus potentially explaining the dwarf phenotype in Jinza 5 (Table 1). Notably, comparative analysis reveals Sanchisan's (~1.2 m) plant height is significantly shorter than typical “I‐dwarf genotype” germplasms (> 2 m), implying the presence of additional dwarfing factors beyond the characterized *dw4* locus. The “II‐dwarf genotype” breeding strategy involves the use of “II‐dwarf genotype” restorer lines such as Jinliang 5 (*Dw1Dw2dw3dw4*), demonstrating strategic gene pyramiding (Liang and Bi, 2014; Wang et al., 2024b). Regarding the “III‑dwarf genotype” varieties, the major genotypes display *Dw2* dominance (*dw1Dw2dw3dw4*) such as Tx3197A, Tx623A, 7051A, and SX605A, while some genotypes exhibit *Dw1* as the dominant gene (*Dw1dw2dw3dw4*) such as A2V4A and SX44A (Multani et al., 2003; Li et al., 2011; Wang et al., 2024b) (Table 1). To date, three dwarf gene loci including *dw1*, *dw2* and *dw3* have been characterized in sorghum.

**Table 1:** Information on the representative Chinese hybrid sorghum varieties

Hybrid sorghum varieties	Parental lines and their genotype (female parent × male parent)	Key genes employed
Jinza 5	Tx3197A (*dw1Dw2dw3dw4*) × Sanchisan (*Dw1Dw2Dw3dw4*)	*dw4*
Jinza 4	Tx3197A (*dw1Dw2dw3dw4*) × Jinliang 5 (*Dw1Dw2dw3dw4*)	*dw3dw4*
Jinzhong 405	7501A (*dw1Dw2dw3dw4*) × Jinliang 5 (*Dw1Dw2dw3dw4*)	*dw3dw4*
Jinza 12	A2V4A (*Dw1dw2dw3dw4*) × 1383‐2 (*Dw1Dw2dw3dw4*)	*dw3dw4*
Jinza 22	A2SX44A (*Dw1dw2dw3dw4*) × SXR‐30 (*Dw1Dw2dw3dw4*)	*dw3dw4*
Jinza 101	A2SX44A (*Dw1dw2dw3dw4*) × 363C/2691 (*Dw1Dw2dw3dw4*)	*dw3dw4*
Jinza 33	SX605A (*dw1Dw2dw3dw4*) × N133 (*Dw1Dw2dw3dw4*)	*dw3dw4*
Jinza 34	SX605A (*dw1Dw2dw3dw4*) × SX861 (*dw1Dw2dw3dw4*)	*dw1dw3dw4*

**Figure 4:**
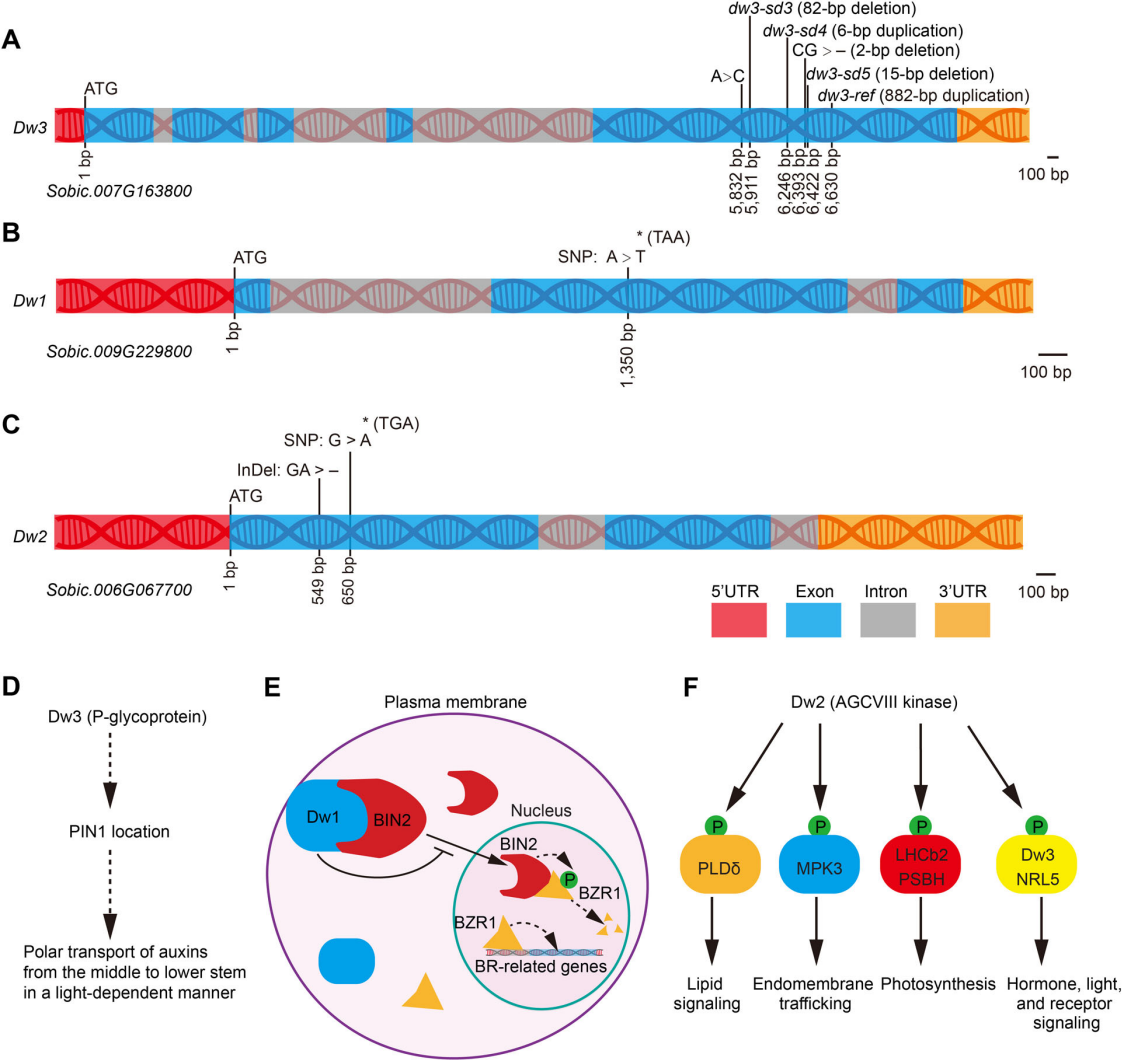
**Detected alleles of the** ***Dw3***, ***Dw1***, **and** ***Dw2*** **genes in different sorghum variants** **(A–C)** Six alleles **(A)**, one allele **(B)** and two alleles **(C)** have been detected in *Dw3*, *Dw1*, and *Dw2* loci, respectively. Gene loci are based on the reference genome of *Sorghum bicolor* v5.1. SNP, single‐nucleotide polymorphism; InDel, insertion and deletion. **(D**, **E)** The molecular mechanism model of dwarf genes *Dw3* (**D**), *Dw1* (**E**), and *Dw2* **(F)** in sorghum. LHC, light‐harvesting complex; MPK3, mitogen‐activated protein kinase 3; NRL5, NPH3/root phototropism 2‐like 5; PLD, phospholipase D; PSBH, photosystem II reaction center protein H.

The *Dw3* (*Sobic.007G163800*) encodes a P‐glycoprotein (PGP) within the adenosine triphosphate‐binding cassette (ABC) transporter family, functioning as a polar auxin transporter. Studies demonstrate that impaired Dw3 disrupts light‐dependent polar auxin transport along the stem basipetal axis probably by impacting the localization of auxin‐efflux carrier PIN1 (Multani et al., 2003) (Figure 4D). Molecular characterization reveals an 882‐bp tandem duplication in exon 5 of the *dw3* mutant, resulting in nonfunctional proteins and conferring dwarfism through reduced internode elongation (Multani et al., 2003) (Figure 4A). Three stable mutant alleles (*dw3‐sd3*, *dw3‐sd4*, and *dw3‐sd5*), containing exon 5 indels (82‐bp deletion, 6‐bp duplication, and 15‐bp deletion), have been introgressed into elite lines to control plant height (Diatta‐Holgate et al., 2024). Recent findings further identified novel *Dw3* variants (1‐bp substitution and 2‐bp deletion) with height‐reducing effects (Wang et al., 2024a) (Figure 4A).

Fine mapping localized *Dw1* to a 33‐kb interval on chromosome 9, which encodes a conserved membrane protein. The dwarf allele *dw1* in parent line 80M contains an A‐to‐T nonsense mutation (Sobic.009G229800 exon 2), resulting in a truncated protein (Hilley et al., 2016) (Figure 4B). Functional studies establish Dw1 as a novel brassinosteroid (BR) signaling activator in sorghum through physical interaction with BRASSINOSTEROID INSENSITIVE 2 (BIN2), inhibiting nuclear translocation of this BR signaling repressor, which may allow BRASSINAZOLE RESISTANT 1 (BZR1) to localize to the nucleus, activating the expression of BR‐related genes (Hirano et al., 2017) (Figure 4E). *dw1* mutant plants display characteristic BR‐deficient phenotypes, confirming its essential role in internode elongation via BR pathway regulation (Hirano et al., 2017).

Positional cloning identified *Dw2* as *Sobic.006G067700*, which encodes an AGCVIII protein kinase. The recessive *dw2* allele carries a GA dinucleotide deletion in exon 1, causing a frameshift truncation of the kinase domain (Hilley et al., 2017) (Figure 4C). Mechanistically, the *dw2* mutation suppresses cell proliferation, alters endocytosis dynamics, and modifies cell wall polysaccharide localization. Phosphoproteomic evidence further links Dw2 to lipid signaling and endomembrane trafficking (Oliver et al., 2021) (Figure 4F). Population genomics reveal strong selection signatures at *Dw2* during sorghum improvement, indicating its agronomic importance (Boatwright et al., 2022). Genome‐wide association study (GWAS) analysis positions *Dw4* within a 6.6‐Mb interval on chromosome 6, although the precise molecular characterization remains pending (Boatwright et al., 2022).

In addition to *Dw1/2/3/4*, more plant height genes have been cloned gradually. Utilizing a recombinant inbred line population, a distinct quantitative trait locus (QTL) for plant height, designated *qHT7.1*, was identified in the genomic region adjacent to the known auxin transporter gene *Dw3* (Li et al., 2015). Detailed analyses demonstrated that *qHT7.1* exerts an influence on both the upper and lower plant segments, in contrast with *Dw3*, which affects solely the region below the flag leaf (Li et al., 2015). Another study on bioenergy sorghum identified a plant height gene named *Dw7a*, encoding a MYB transcription factor, as the causal gene underlying a plant height QTL (referred to as *qCL‐7a* in their research). This was achieved by fine mapping the QTL region to a 21‐kb interval harboring a single annotated gene, *Sobic.007G137101* (Hashimoto et al., 2021). However, the causal polymorphism in sorghum and the relationship of *Dw7a* with *qHT7.1* remain uncharacterized. Subsequent studies revealed that *Sobic.007G137101*, which underlies the *qHT7.1* locus and encodes a MYB transcription factor (MYB110), can control internode elongation, cell proliferation, and cell morphology. A 740‐bp transposable element insertion in the intron region of *MYB110* triggered partial mis‐splicing, resulting in a novel transcript containing an extra exon and a premature stop codon, ultimately contributing to reduced plant height (Mu et al., 2024).

In summary, it is proposed that sorghum breeding has exploited mutations in the auxin and BR signaling pathways to reduce plant height. This approach differs from the gibberellin (GA) deficiency strategy employed in rice and wheat dwarf breeding.

## THE DEVELOPMENT OF TWO‐LINE HYBRID IN SORGHUM

The two‐line hybridization system utilizes genic male‐sterility (GMS) mutations regulated by environmental factors such as photoperiod or temperature, enabling fertility conversion through conditional control (Fan and Zhang, 2018; Li et al., 2021; Hou et al., 2022). This approach employs photoperiod‐/thermo‐sensitive genic male sterile (P/TGMS) lines that exhibit male sterility under restrictive environmental conditions (e.g., specific temperature thresholds) and regain fertility under permissive conditions (Figure 2B). Compared with the three‐line system, the two‐line method simplifies hybrid seed production by eliminating the need for a maintainer line. Despite its operational advantages, two‐line hybrids remain less prevalent in sorghum compared with crops such as rice. The development of sorghum two‐line systems began in 1988 with the creation of Xiangnuoliang S‐1, the world's first thermo‐sensitive GMS line, by the Hunan Soil and Fertilizer Research Institute in China (Li et al., 1994). This line exhibits sterility at less than 23.8°C and restores fertility above this threshold. Within the temperature range of fertility conversion, the photoperiod has a certain influence on the fertility of Xiangnuoliang S‐1, manifested as short‐day photoperiod promoting sterility and long‐day photoperiod promoting fertility (Chen et al., 2002). Its successful application produced the two‐line hybrid cultivar Xiangliangyounuoliang 1 through crosses with the restorer line Xiang 10721, which achieved yields up to 8,250 kg/ha (Zhao, 1996). Subsequent advancements included Wenguang Tang's group developing Xiangnuoliang 2S, a GMS line with elevated fertility conversion temperature, which was crossed with the restorer line C4Y721 to create hybrid Xingxiangliang 2 (Tang et al., 2007). Further progress was made in 2000 with the cultivation of Jiuyinuoliang S‐1, which was derived from the progeny of Xiangnuoliang S‐1 and exhibited a higher fertility‐conversion temperature of 28°C (Chen et al., 2002). Despite these achievements and operational advantages, two‐line sorghum hybrids occupy limited cultivation areas due to two primary constraints: (i) Instability of male sterility under field conditions caused by temperature fluctuations, and (ii) incomplete fertility restoration during self‐pollination phases, which reduces the seed efficiency of production.

The molecular mechanisms underlying two‐line systems remain poorly characterized. Preliminary investigations revealed a unique 92‐kDa protein present specifically in the sterile phase of Xiangnuoliang S‐1, alongside elevated peroxidase activity in fertile plants, suggesting potential biochemical markers for fertility conversion (Li et al., 1994).

## THE DEVELOPMENT OF ONE‐LINE SORGHUM BREEDING

The one‐line breeding strategy aims to perpetuate heterosis through apomixis, which is an asexual reproductive mechanism that enables the formation of seeds without fertilization (Figure 2C). Despite its potential for revolutionizing crop breeding, functional apomixis remains rare in major agricultural crops. The initial evidence of apomictic reproduction in sorghum emerged in 1968 with the identification of apomixis traits in the sorghum line R473 (Rao and Narayana, 1968). Subsequent studies have confirmed the development of aposporous and diplosporous embryo sac across multiple sorghum genotypes (Hanna et al., 1970; Wu et al., 1994; Elkonin et al., 1995).

Following the earlier international efforts, Chinese researchers have made significant contributions since the 1990s to the establishment of the one‐line sorghum breeding system. In 1991, Fuyao Zhang et al. (1991) first reported the presence of apomictic reproduction in the sorghum line 296B. In 1994, Tiantang Niu successfully bred the facultative apomictic lines of sorghum, SSA‐1 and 296B, with their apomixis frequencies reaching 50% and 21%, respectively, achieving a groundbreaking achievement in this research area in China (Niu et al., 1994). Notably, 296B and R473 have special compatibility characteristics. When these two lines are crossed and the resulting hybrid is backcrossed with 296B, offspring with essentially fixed heterosis can be obtained, while backcrossing with R473 can only partially fix the heterosis (Niu et al., 1994). However, the excessively low apomixis frequency remains a major obstacle restricting the fixation of heterosis in crops.

The underlying mechanisms of apomixis in sorghum remain largely unknown. The apomictic lines of sorghum mainly include four types: apospory, diplospory, adventitious embryony and female parthenogenesis. Apospory is the main type of apomixis in sorghum. The aposporous initial cells originate from nucellar cells and form four‐nucleate or eight‐nucleate embryo sacs through two to three mitotic divisions. Sorghum lines R473 and 296B are both of the aposporous type, and SSA‐1 can also undergo apospory (Ping, et al., 2009). A diplosporous reproductive mode was first discovered in the sorghum line SSA‐1, where the megaspore mother cell undergoes direct mitosis without meiosis to form an 8‐nucleate embryo sac (Niu et al., 1994). The functional components of apomixis have been observed in the grain sorghum line AS‐3 (Belyaeva et al., 2021). The genetic locus controlling apomixis, called apospory‐specific genomic region (ASGR), has been detected in *Pennisetum squamulatum* and *Cenchrus ciliaris*. However, sequence analysis revealed that the ASGR does not exhibit large‐scale synteny with either rice or sorghum genomes (Conner et al., 2008).

## MOLECULAR DESIGN BREEDING OF SORGHUM

With the advancement of molecular biotechnology, molecular design breeding, including molecular marker‐assisted breeding and gene‐edited breeding, has provided new approaches for sorghum improvement in China. Chinese sorghum researchers have developed molecular markers for disease and pest resistance in sorghum such as aphid resistance and head smut resistance, which can be used in marker‐assisted breeding (Li et al., 2006; Lu et al., 2009). Chinese researchers have also identified that *qGW1*, a major locus influencing grain weight, has been fine mapped to a 101‐kb region on the short arm of chromosome 1 in sorghum. Sobic.001G038900 was identified as the candidate gene within this interval (Han et al., 2015). Additionally, molecular markers have been developed for known variant sites in the height‐related genes *Dw1*, *Dw2*, and *Dw3* (Wang et al., 2025). These markers are of great significance for sorghum breeding. In recent years, gene‐editing technologies have been increasingly employed in Chinese sorghum breeding. Qi Xie's research team, from IGDB, CAS, has successfully created a new variety of fragrant sorghum by knocking out the *SbBADH2* gene using CRISPR/Cas9 gene‐editing technology (Zhang et al., 2022). A major locus particularly related to alkaline‐salt sensitivity, *Alkaline Tolerance 1* (*AT1*), was also identified by this research team through GWAS (Zhang et al., 2023). Knockout of the *AT1* gene increases the alkali tolerance of sorghum, millet, rice, and maize. *AT1* encodes an atypical G protein γ subunit, which affects the phosphorylation of aquaporins, thereby regulating the distribution of hydrogen peroxide (H_2_O_2_) (Zhang et al., 2023). Designing the knockout of *AT1* homologous genes or selecting their naturally occurring nonfunctional alleles can improve crop yields in saline‐alkali soils. A recent study identified two ABCG transporters of strigolactones (SLs), SbSLT1 and SbSLT2, through transcriptomic and functional analyses of sorghum under phosphate deficiency or treated with the SL GR24^5DS^ (Shi et al., 2025). CRISPR/Cas9‐mediated knockout of *SbSLT1* and *SbSLT2* significantly reduced SL contents in root exudates of the knockout mutants. Consequently, the germination rate of *Striga* seeds treated with these exudates decreased significantly. Field experiments revealed that parasitism rates in *SbSLT1* ^*KO*^ *SbSLT2* ^*KO*^ were reduced by 67%–94%, while sorghum yield losses were simultaneously mitigated by 49%–52% (Shi et al., 2025).

## FUTURE PROSPECTS OF SORGHUM BREEDING

**Figure 5:**
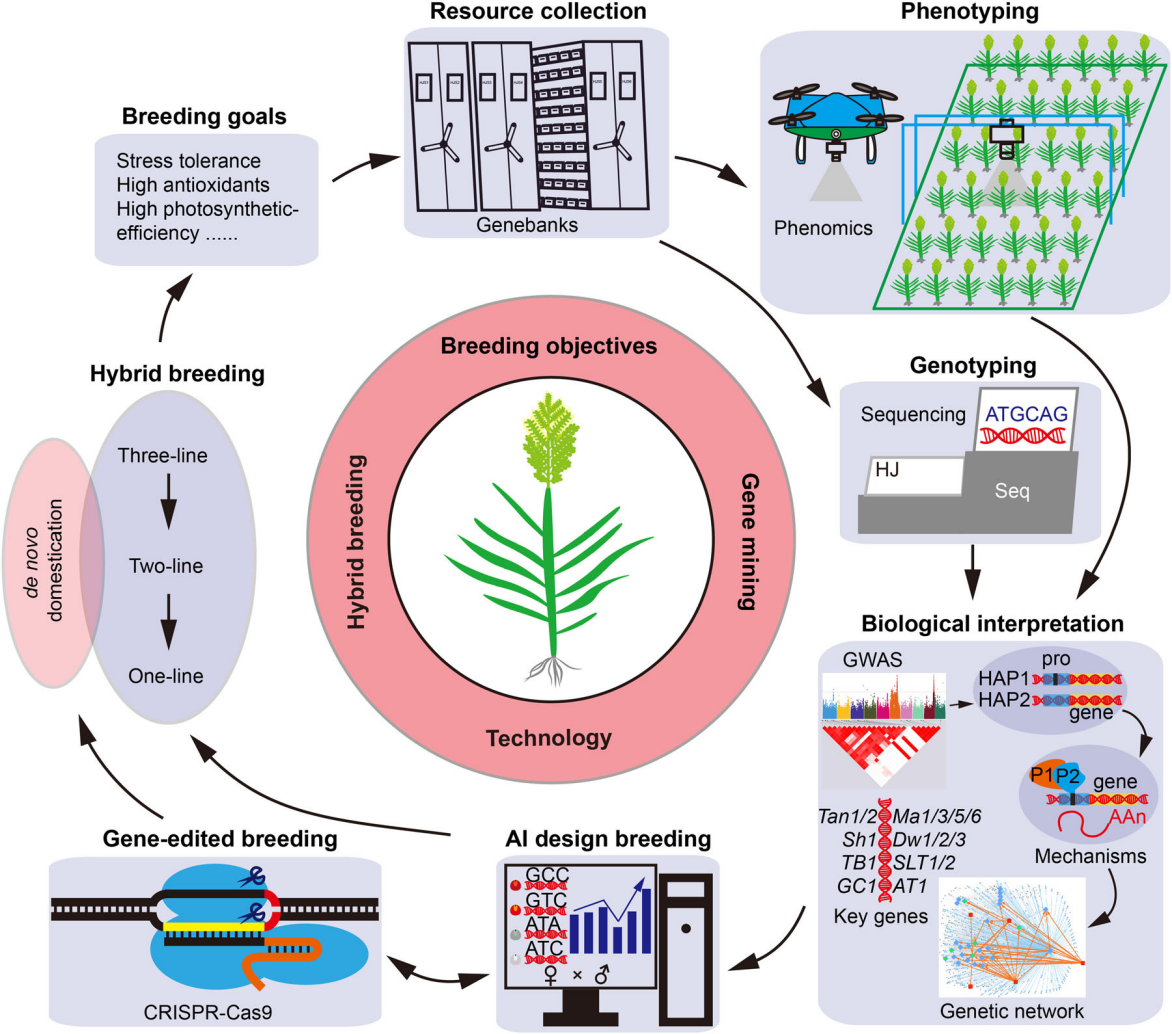
Future sorghum breeding The breeding goals of sorghum will be more diversified to meet the changing market demands and the requirements of sustainable agricultural development. The rich germplasm resources preserved in the gene bank, such as wild species, lay a solid foundation for molecular design breeding. The process of elucidating the mechanisms of beneficial genes, such as *Tan1/2*, *Ma1/3/5/6*, *Sh1*, *Dw1/2/3*, *TB1*, *AT1*, S*LT1/2*, so forth, can facilitate molecular design breeding (Ge et al., 2023; Zhang et al., 2023; Shi et al., 2025). High‐throughput phenotyping, genotyping, gene editing (e.g., CRISPR/Cas9), and AI‐based design breeding provide reliable approaches for molecular design breeding. Advancements in these aspects hold great promise for promoting the shift from the three‐line to the one‐line system and realizing *de novo* domestication.

Dwarf hybrid breeding has been instrumental in driving sorghum production in China. The progressive innovation shift from the three‐line method to the two‐line method and ultimately toward the one‐line method represents a strategic paradigm shift (Figure 5). Although the established three‐line method demonstrates superiority in the utilization of heterosis, it has some limitations, including complex seed production procedures and genetic base constraints. The two‐line method simplifies the seed production via P/TGMS lines, yet its stability is susceptible to environmental fluctuations. Integration of molecular marker‐assisted selection (MAS) with smart environmental control technologies could improve the ecological adaptability of sterile lines. The one‐line system, aiming to perpetuate heterosis through apomixis, remains largely theoretical. However, emerging technologies such as CRISPR/Cas9‐mediated gene editing and synthetic biology offer promising avenues for designing apomixis‐associated gene modules, potentially overcoming current limitations in multi‐generation utilization of hybrids (Zheng et al., 2024).

In recent years, with the development of society and the improvement of people's living standards, sorghum has shifted from being mainly used as a staple food to being used for processing, feeding livestock, and other purposes. Breeding researchers have explored new directions for the application of hybrid sorghum, such as sorghum varieties suitable for winemaking and mechanized harvesting. However, there is still a considerable gap between the current hybrid sorghum breeding and market demands. In the future, with the ever‐changing market demands and the imperatives of sustainable agricultural development, the breeding objectives of sorghum are bound to become more diversified and specifically tailored to industrial needs (Figure 5). Confronted with the global imperatives of food security and carbon neutrality, future sorghum breeding needs to focus on the dual goals of “green and efficient” and “functional diversification”. On the one hand, by modulating metabolic pathways and enhancing C_4_ photosynthetic efficiency, varieties of energy sorghum and forage sorghum with double biomass can be developed. This will in turn promote the development of the bio‐economy and the upgrade of the livestock industry. On the other hand, functional sorghum varieties with high antioxidant content (e.g., phenols and anthocyanins) can be developed to meet the demands of the healthy food market.

Future sorghum breeding will be characterized by multidisciplinary technological integration (Figure 5). First, in line with breeding objectives, strengthen the collection of wild sorghum and local germplasm resources, and expand the germplasm repository through distant hybridization and mutation breeding. Second, artificial intelligence (AI)‐enhanced phenomics will accelerate high‐throughput phenotypic screening of populations, enabling the construction of genotype‐phenotype‐environment interaction models to optimize parental selection (Fu et al., 2025). Third, relying on phenotypic and genomic data, the GWAS in combination with molecular biotechnology will unlock the potential to explore gene functions and regulatory mechanisms. Furthermore, CRISPR‐based genetic background purification will transcend the germplasm bottlenecks of conventional hybridization, fostering cultivars suited for marginal lands and extreme climates (Iswanto et al., 2025). In addition, AI‐powered prediction models integrated with genome‐wide selection (GS) leverage machine learning algorithms to analyze multidimensional genomic and phenotypic datasets, facilitating rapid identification of superior hybrid combinations and drastically reducing breeding cycle duration (Fu et al., 2025). Moreover, with recent progress in genomics and molecular biology, as well as the rapid update of advanced scientific research equipment and agricultural machinery, a new breeding strategy, *de novo* domestication, has become a hot topic in modern crop breeding (Yu and Li, 2022; Zhang et al., 2023a). *De novo* domestication is an innovative breeding strategy involving the rapid domestication of wild plants by redesigning agronomic traits and introducing domestication genes to meet diverse human needs for developing new crop varieties (Yu et al., 2021; Zhang et al., 2023b). Realizing the ingenious integration of *de novo* domestication and cross‐breeding in sorghum breeding is conducive to exploring new and excellent gene resources, cultivating diversified target‐oriented varieties, and significantly improving breeding efficiency. Overall, by leveraging multidisciplinary integration and technological innovation, sorghum hybrid breeding is poised to transition from being “experience driven” to “intelligently designed driven”, thus providing a core seed‐source guarantee for the sustainable development of global agriculture.

## GENERATIVE AI USAGE STATEMENT

During the preparation of this work, the authors utilized (doubao, https://www.doubao.com/) for the purpose of improving the language of this manuscript. After using this tool, the authors carefully reviewed and revised the content where necessary, and take full responsibility for the final version of the publication.

## CONFLICTS OF INTEREST

The authors declare no conflicts of interest.

## AUTHOR CONTRIBUTIONS

Z.K. conceived the manuscript; X.M. and L.L. drafted the manuscript; X.M., L.L., L.J., and Q.Q. revised the manuscript. The authors read and approved the final manuscript.
